# Estimation of Potential Distribution during Crevice Corrosion through Analysis of *I–V* Curves Obtained by LAPS

**DOI:** 10.3390/s20102873

**Published:** 2020-05-19

**Authors:** Kiyomi Nose, Ko-ichiro Miyamoto, Tatsuo Yoshinobu

**Affiliations:** 1 Material Performance Solution Center, Nippon Steel Technology Corporation, 20-1, Shintomi, Futtu 293-0011, Japan; 2 Department of Electronic Engineering, Tohoku University, 6-6, Aza-Aoba, Aramaki, Aoba-ku, Sendai 980-8579, Japan; k-miya@ecei.tohoku.ac.jp (K.-i.M.); nov@ecei.tohoku.ac.jp (T.Y.); 3 Department of Biomedical Engineering, Tohoku University, 6-6, Aza-Aoba, Aramaki, Aoba-ku, Sendai 980-8579, Japan

**Keywords:** light-addressable potentiometric sensor, LAPS, crevice corrosion, potential distribution, crevice gap

## Abstract

Crevice corrosion is a type of local corrosion which occurs when a metal surface is confined in a narrow gap on the order of 10 μm filled with a solution. Because of the inaccessible geometry, experimental methods to analyze the inner space of the crevice have been limited. In this study, a light-addressable potentiometric sensor (LAPS) was employed to estimate the potential distribution inside the crevice owing to the IR drop by the anodic current flowing out of the structure. Before crevice corrosion, the *I–V* curve of the LAPS showed a potential shift, depending on the distance from the perimeter. The shift reflected the potential distribution due to the IR drop by the anodic current flowing out of the crevice. After crevice corrosion, the corrosion current increased exponentially, and a local pH change was detected where the corrosion was initiated. A simple model of the IR drop was used to calculate the crevice gap, which was 12 μm—a value close to the previously reported values. Thus, the simultaneous measurement of the *I–V* curves obtained using a LAPS during potentiostatic electrolysis could be applied as a new method for estimating the potential distribution in the crevice.

## 1. Introduction

Stainless steel is a highly corrosion-resistant alloy; however, it corrodes under certain circumstances. Crevice corrosion [1,2,3,4,5,6] is a type of localized corrosion of stainless steel which occurs in the presence of chlorides, where the surface is confined in a narrow gap on the order of 10 μm. It occurs and develops in accordance with the following steps [1,4,5,6]: (1) consumption of the dissolved oxygen in the crevice, (2) formation of a differential ventilation battery, (3) increase in chloride ion concentration, and (4) lowering of pH owing to a hydrolysis reaction of eluted metal ions. Crevice corrosion depends strongly on the geometry, which affects the transport of ions inside and outside of the crevice.

The crevice corrosion resistance of stainless steel is usually evaluated by determining the critical crevice temperature (CCT) in an immersion test (in accordance with ASTM G48 Method F) [7], measuring the crevice corrosion repassivation potential [8], or conducting an electrochemical corrosion test [9,10,11,12] using a potentiostatic test. In electrochemical corrosion tests, an IR drop occurs due to the direct current flowing from the inside of the crevice to the outside. This IR drop becomes larger when the crevice gap is smaller, and when the conductivity of the solution in the crevice is lower. The IR drop results in a potential distribution and therefore a non-uniform biasing condition inside the crevice, depending on the distance from the perimeter. For the potential distribution inside a crevice during a potentiostatic test, a study based on numerical simulation has been reported [13]. However, there have been few experimental studies in which the potential distribution was measured.

In this study, we employed a light-addressable potentiometric sensor (LAPS) [14] to measure the potential distribution inside a crevice. The LAPS is a semiconductor-based chemical sensor in which a light beam generates a photocurrent signal depending on the local potential of the sensor surface at the illuminated position. By using a pH-sensitive Si_3_N_4_ surface and a scanning light beam, a LAPS can measure the pH distribution of the solution in contact with the sensor surface [15,16]. A LAPS was also applied to the visualization of pH distribution during crevice corrosion [17,18,19] in a metal/sensor crevice structure formed by mounting a metal specimen directly on the sensor surface. We propose to use the same setup to measure the potential distribution rather than the pH distribution inside a crevice and to estimate the crevice gap, where the IR drop is superimposed on the bias voltage applied to the LAPS.

## 2. Experimental Methods

### 2.1. Measurement Setup

**Figure 1:**
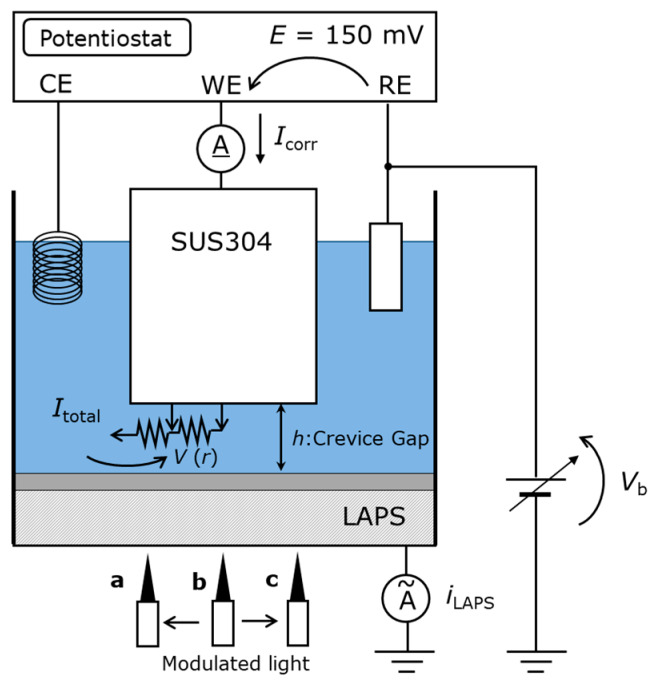
Setup for simultaneously performing the potentiostatic test and light-addressable potentiometric sensor (LAPS) measurement. RE, WE, and CE indicate the reference electrode, working electrode, and counter electrode, respectively. In this experiment, an Ag/AgCl_(3M NaCl)_ electrode, a SUS304 specimen, and a platinum wire were used as the RE, WE, and CE, respectively. The potentiostat was used to apply a controlled potential to the specimen and to monitor the corrosion current *I*_corr_. The points of illumination **a**, **b**, and **c** correspond to the surface locations shown in the top-right image of Figure 2.

Figure 1 shows the configuration of the equipment used for potentiostatic test and the *I–V* curve measurement with the LAPS. The sensor plate was fabricated by depositing the SiO_2_ and Si_3_N_4_ films on an n-type Si substrate, as described in a previous study [16]. A laser beam with a wavelength of 830 nm modulated at a frequency of 2500 Hz illuminated the rear surface of the sensor plate to generate an alternating photocurrent signal (hereinafter expressed as *I*_p_). As the LAPS surface was insulated, only the alternating current flowed through the LAPS sensor plate. Most of the direct current (*I*_corr_) returned to the counter electrode (CE).

A cylindrical bar made of SUS304 (0.068% carbon, 1.84% manganese, 0.029% phosphorus, 0.027% sulfur, 8.11% nickel, and 18.65% chromium) with a diameter of 12 mm and a height of 50 mm was used as the specimen. This was mounted on the sensor surface to form a metal/sensor crevice structure.

The specimen was subjected in advance to ultrasonic cleaning in acetone and passivated in 30% HNO_3_ solution at 50 °C. Two different solutions prepared by diluting artificial seawater (ASW) were used as test solutions. Table 1 lists the specific conductivity and pH of each solution. Here, 5 mL of test solution was poured into the measurement cell, the bottom of which was the sensor surface. To remove the oxide film on the surface of the specimen, the crevice-forming plane of the specimen was polished with #1000 wet sandpaper immediately prior to the experiment. Polishing was completed when the surface did not repel water. The finished surface was mirror-like, which guaranteed fair reproducibility of the corrosion experiment. A large difference in roughness would affect the corrosion due to differences in the surface area and the crevice gap.

### 2.2. Crevice Corrosion Test 

After the specimen was mounted on the sensor surface, the spontaneous potential of SUS304 was monitored. When the potential value became −200 ± 10 mV, the potentiostatic test and *I–V* curve measurement were started simultaneously. The potentiostatic test was performed at 150 mV vs. Ag/AgCl_(3M NaCl)_ (hereinafter expressed by the millivolt value only), and a platinum electrode was used as the CE.

### 2.3. I–V Curve Measurement 

During the potentiostatic test, the alternating photocurrent signal *I*_p_ of LAPS was repeatedly measured according to the following scheme: first, the bias voltage applied to the LAPS was set at −1400 mV, and the laser beam was moved along the diameter of the SUS304 piece. During this scan, *I*_p_ was recorded as a function of the position on the diameter with a constant spacing of 400 μm. The same diameter was repeatedly scanned while changing the bias voltage from −1400 to −1000 mV with an interval of 50 mV, and from −1000 to 0 mV with an interval of 20 mV. After completing these scans, the values of *I*_p_ measured at the same position were gathered to construct an *I–V* curve in the range of −1400 to 0 mV, and the bias voltage corresponding to the inflection point of this *I–V* curve was calculated as an indicator of the potential change at that position. This scan was repeated every 145 s throughout the potentiostatic test.

## 3. Results and Discussion

### 3.1. Shift of I–V Curves Obtained by the LAPS during Crevice Corrosion

**Figure 2:**
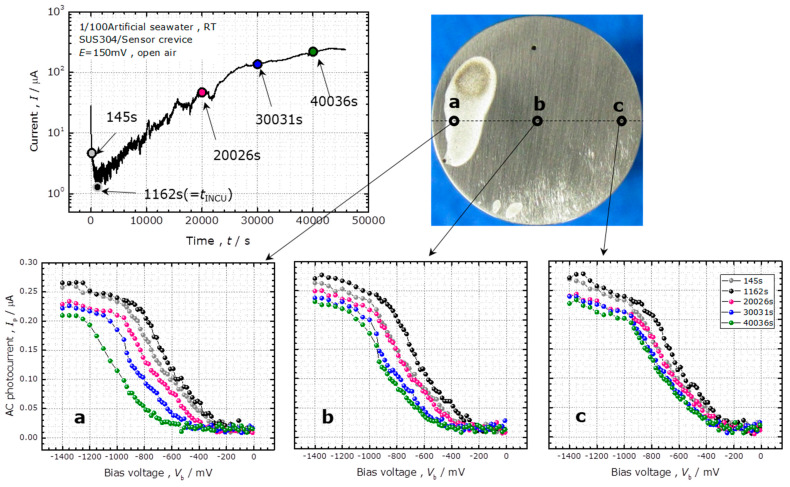
(**Top left**) The temporal change of the corrosion current during the potentiostatic test of a SUS304 specimen at *E* = 150 mV in 1/100 ASW. (**Top right**) Optical photograph of the corroded surface after 48,132 s of the corrosion test. (**Bottom**) I–V curves measured at different points a, b, and **c**.

The results of a corrosion test in 1/100 ASW are shown in Figure 2. The temporal change of the corrosion current and the *I–V* curves at different positions are presented together with an optical photograph of the corroded surface after the corrosion test. Point **a** is located within the corroded area; point **b** is the center of the specimen, and point **c** is near the right edge of the specimen. Timestamps of 145, 1162, 20,026, 30,031, and 40,036 s are indicated on the curve of the corrosion current, and the *I–V* curves acquired at these points of time are shown below.

The temporal change of the corrosion current shows that the incubation time of crevice corrosion (*t*_INCU_) [12] was approximately 1162 s, after which the corrosion current increased exponentially with time. At point **a**, the *I–V* curve moved rightward (i.e., towards higher bias voltages) after the start of the potentiostatic test until *t*_INCU_. Then, the *I–V* curve moved leftward with the progress of corrosion. At point **b**, the rightward shift until *t*_INCU_ was larger than that of point **a**, and the leftward shift after *t*_INCU_ was smaller than that of point **a**. At point **c**, the rightward shift was similar to that of point **a**, and the leftward shift after the *t*_INCU_ was smaller. For interpretation of these results, the shift of the *I–V* curve was quantitatively analyzed as shown in the following sections. 

### 3.2. Analysis of the Shift of I–V Curves

**Figure 3:**
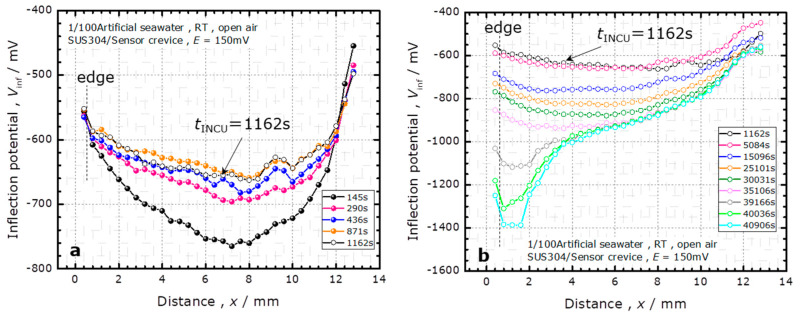
The temporal change of the spatial distribution of the inflection points of *I–V* curves (*V*_inf_) in the course of the potentiostatic test at *E* = 150 mV in 1/100 ASW. (**a**) The early stage before the occurrence of crevice corrosion; (**b**) The later stage after the occurrence of crevice corrosion.

To analyze the shift of the *I–V* curves during the potentiostatic test, the inflection point near the middle of the transition region of each *I–V* curve (hereinafter expressed as *V*_inf_) was calculated. Figure 3 shows the results for the corrosion in 1/100 ASW. The temporal change of the spatial distribution of *V*_inf_ is shown separately in Figure 3a,b, for the early stage before *t*_INCU_ and the later stage after *t*_INCU_, respectively. 

In Figure 3a, the spatial distribution of *V*_inf_ has a valley shape. *V*_inf_ decreases as the measurement point goes further from the edge of the specimen and reaches a minimum at approximately the center of the specimen. With increasing time during the potentiostatic test, this valley becomes shallower until the occurrence of corrosion at *t*_INCU_. The effective bias applied to the EIS (electrolyte–insulator–semiconductor) structure of a LAPS is the total of the externally applied bias (*V*_b_), the change of the Nernst potential sensitive to pH, and the IR drop owing to the DC current flowing out of the crevice. As the pH remains the same, the shift of the *I–V* curve is a direct measure of the IR drop. The pH change remains negligible at the early stage before *t*_INCU_; therefore, the spatial distribution of *V*_inf_ should be attributed to the potential distribution owing to the IR drop. As the corrosion current decreased until *t*_INCU_, the IR drop became smaller, and the valley became shallower.

Here, we consider the potential distribution *V*(*r*) owing to the IR drop in the crevice. Using the potential outside the crevice as a reference, the *V*(*r*) was 0 at the edge of the specimen. When the specimen was anodically polarized, a direct current flowed out of the crevice, and *V*(*r*) became higher inside the crevice. In a LAPS measurement, a bias voltage *V*_b_ is defined as the potential of the Ag/AgCl_(3M NaCl)_ reference electrode with respect to the potential of the sensor substrate. Under the potential distribution in the crevice, the effective bias applied to the local position on the sensor surface was *V*_b_ + *V*(*r*), which explains the spatial distribution of the inflection potential of *V*_inf_ shown in Figure 3a. The higher the potential *V*(*r*), the larger the leftward shift of the *I–V* curve obtained by the LAPS. Simultaneously, when a constant potential of *E* = 150 mV with respect to the reference electrode was applied to the specimen, the effective polarization at the local position on the SUS304 surface was decreased to 150 mV – *V*(*r*).

After crevice corrosion at *t*_INCU_, the inflection potential *V*_inf_ decreased, as shown in Figure 3b, suggesting acidification of the solution in the crevice by the reaction:

(1) $$M^{n+}+nH_{2}O\to M{\left(OH\right)}_{n}+nH^{+}$$

where M*^n^*^+^ denotes eluted metal ions. Near the left edge of the specimen, where the SUS304 surface was corroded, the *V*_inf_ sharply decreased after 39,166 s.

### 3.3. Estimation of the Potential Distribution inside the Crevice

Based on the discussion above, the potential distribution inside the crevice *V*(*r*) was equal to the leftward shift of the *I–V* curve, provided that the pH change is negligible. The distribution of the *V*_inf_ at *t* = 145 s was considered directly after the start of the potentiostatic test. We redefined the position *r* with respect to the center of the specimen; *r* = 0 is the center, and *r* = ± *R* is the edge of the specimen. If we take the potential at the edge as a reference, *V*(*r*) is given by:

(2) $$V\left(r\right)=V_{\inf}\left(R\right)-V_{\inf}\left(r\right)$$

**Figure 4:**
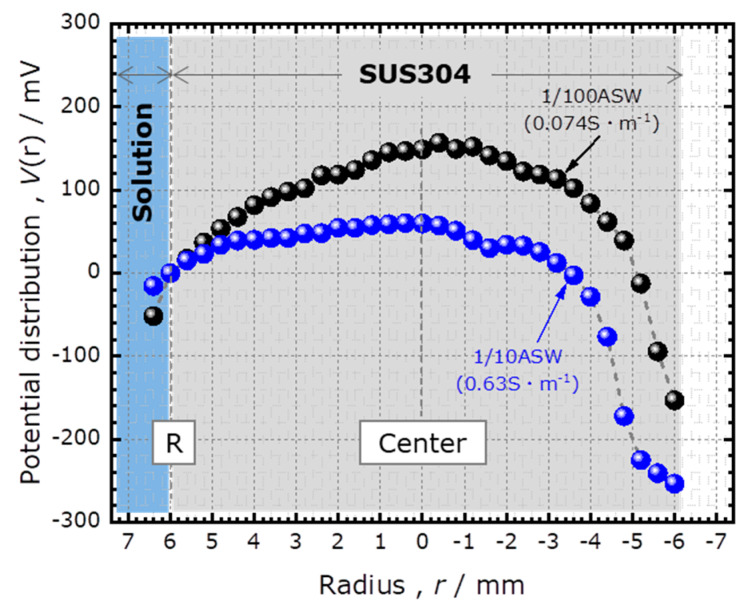
The calculated potential distribution inside the crevice immediately after the start of the potentiostatic test at *E* = 150 mV in 1/100 ASW and 1/10 ASW.

Figure 4 shows the potential distribution *V*(*r*) immediately after the start of the potentiostatic test in 1/100 ASW and 1/10 ASW. The specific conductivities of these solutions were 0.074 and 0.63 Sm^−1^, respectively. In both cases, the potential became higher with increased distance from the edge. The smaller IR drop for 1/10 ASW was consistent with its higher conductivity. The reason for the asymmetry of the curve, especially near the right edge, is unknown; it could be due to a slight tilt of the specimen or convection of the solution.

Figure 4 shows that the value of the *V*(*r*) at the center was 149 mV and 60 mV for 1/100 ASW and 1/10 ASW, respectively. When the specimen was biased at *E* = +150 mV vs. Ag/AgCl_(3M NaCl)_ reference electrode, the effective polarization voltage at the center of the specimen was estimated to be +1 and +90 mV, respectively. Considering that the spontaneous potential of SUS304 at the start of the potentiostatic test was approximately −200 mV, polarization at +1 and +90 mV was still in the anodic region. Therefore, the entire surface of SUS304 in the crevice was anodically polarized.

### 3.4. Estimation of the Crevice Gap

In the crevice corrosion experiment, the SUS304 specimen was placed directly on the sensor surface by its own weight, and the crevice gap remained unknown as it was determined by the surface roughness. Here, we estimated the crevice gap using a simple model, based on the potential distribution obtained in the previous section and the externally measurable total current.

**Figure 5:**
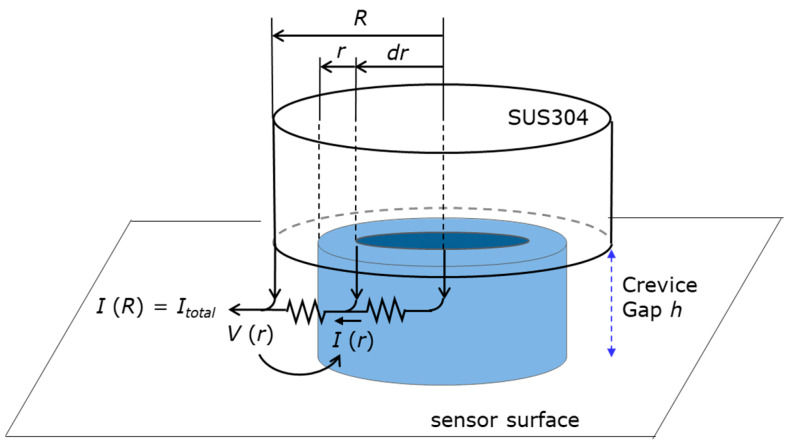
The model used for estimation of the crevice gap.

We assumed a uniform crevice gap *h* and considered a hollow cylinder in the crevice with radius *r*, thickness *dr,* and height *h,* as shown in Figure 5. When the specific conductivity of the solution in the crevice is *σ*, the resistance between the inner and outer walls of the cylinder filled by the solution is expressed as:

(3) $$\frac{dr}{2\pi rh\sigma }$$

We define *I*(*r*) as the current flowing outward from the outer wall of this hollow cylinder. The IR drop between *r* and *r* + *dr* is given by:

(4) $$dV=-\frac{dr}{2\pi rh\sigma }\cdot I\left(r\right).$$

We also define *j*(*r*) as the density of the current flowing out of the crevice-forming plane of the specimen into the crevice. The infinitesimal current (*dI*) flowing into the hollow cylinder is then given by:

(5) dI=j(r)·2πrdr.

Combining Equations (4) and (5), we obtain:

(6) $$j\left(r\right)=\frac{1}{2\pi r}\cdot \frac{dI}{dr}=-h\sigma \left[\frac{d^{2}V}{dr^{2}}+\frac{1}{r}\frac{dV}{dr}\right].$$

By fitting the experimentally obtained *V*(*r*) with a parabola,

(7) $$V\left(r\right)=A-Br^{2},$$

we can determine the fitting parameters *A* and *B*, which will give:

(8) $$\frac{d^{2}V}{dr^{2}}+\frac{1}{r}\frac{dV}{dr}=-4B.$$

Therefore, an approximation of *V*(*r*) with a parabola implies that the current density in Equation (6) is independent of *r*, and is given by:

(9) j=4Bhσ.

**Figure 6:**
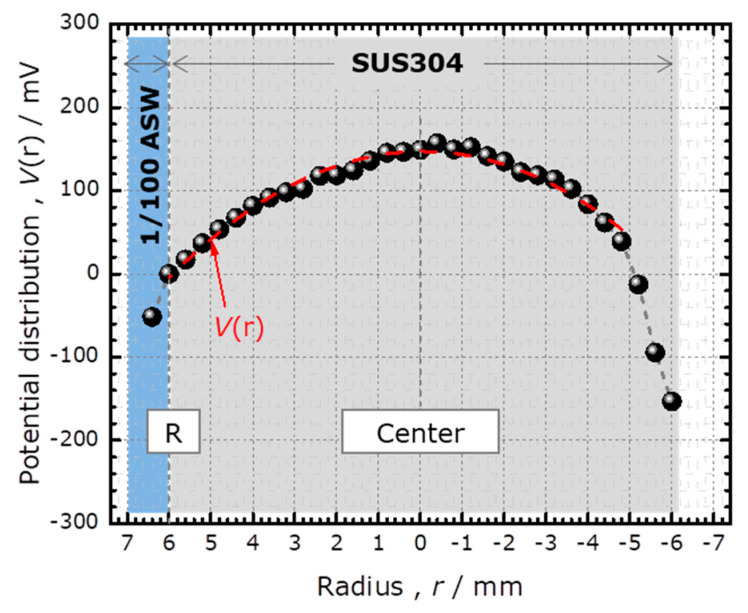
The fitting of the experimentally obtained potential distribution *V*(*r*) with a parabola.

The polarization current *I*_total_ (passive current) flowing into the crevice is then given by:

(10) $$I_{\mathrm{total}}=4Bh\sigma \pi R^{2},$$

which can be used to determine the value of *h*. We applied this analysis to the experimentally obtained potential distribution *V*(*r*) directly after the start of the potentiostatic test in 1/100 ASW, which is shown in Figure 4. Fitting this curve with a parabola as shown in Figure 6, the values of *A* and *B* were determined to be 0.147 V and 4140 Vm^−2^, respectively. As shown in the top-left image of Figure 2, the corrosion current measured at 145 s was 4.6 μA, which included the current flowing into the crevice (*I*_total_) and the current flowing out of the side wall of the specimen contacting the solution. Considering the ratio of the surface areas at the bottom and on the side wall of the specimen, where SUS304 contacted the solution, *I*_total_ was estimated to be 1.7 μA. Using these values as well as *R* = 6 mm and *σ* = 0.074 Sm^−1^ in Equation (10), the crevice gap *h* was estimated to be 12 μm. This value is similar to the previously reported values of 6 to 12 μm obtained by measuring the weight of ethanol filling the crevice gap between an SUS304 surface and a quartz glass rod [11].

## 4. Conclusions

In this study, a LAPS was employed to analyze the potential distribution inside a narrow gap during crevice corrosion of SUS304. A potentiostatic test and *I–V* curve measurement by a LAPS were simultaneously performed. The setup included an SUS304 specimen placed directly on the LAPS surface with a narrow gap filled with ASW. Before the crevice corrosion at *t*_INCU_, the plot of the inflection point of the *I–V* curve showed a valley shape across the crevice, reflecting the potential distribution due to the IR drop by the anodic current flowing out of the crevice. After *t*_INCU_, the corrosion current increased exponentially, and a local change of the inflection point due to the lowering pH was observed at the position where the surface was corroded. A simple model of the IR drop was proposed to describe the potential distribution before *t*_INCU_, which was used to estimate the crevice gap by fitting the experimental data. The estimated gap of 12 μm was similar to the previously reported values obtained by measuring the weight of the liquid filling the gap.

## Figures and Tables

**Table 1:** Specific conductivity and pH of each test solution.

Parameter	1/100 Artificial Seawater	1/10 Artificial Seawater
Specific conductivity (Sm^−1^)	0.074	0.63
pH	6.35	7.21
